# Contrasting motivational orientation and evaluative coding accounts: on the need to differentiate the effectors of approach/avoidance responses

**DOI:** 10.3389/fpsyg.2015.00563

**Published:** 2015-05-01

**Authors:** Julia Kozlik, Roland Neumann, Ljubica Lozo

**Affiliations:** ^1^Department of Psychology, University of Trier, Trier, Germany; ^2^Department of Psychology, University of Würzburg, Würzburg, Germany

**Keywords:** emotional responses, approach and avoidance, affective S–R compatibility, facial muscle contractions, theory of event coding

## Abstract

Several emotion theorists suggest that valenced stimuli automatically trigger motivational orientations and thereby facilitate corresponding behavior. Positive stimuli were thought to activate approach motivational circuits which in turn primed approach-related behavioral tendencies whereas negative stimuli were supposed to activate avoidance motivational circuits so that avoidance-related behavioral tendencies were primed (*motivational orientation account*). However, recent research suggests that typically observed affective stimulus–response compatibility phenomena might be entirely explained in terms of theories accounting for mechanisms of general action control instead of assuming motivational orientations to mediate the effects (*evaluative coding account*). In what follows, we explore to what extent this notion is applicable. We present literature suggesting that evaluative coding mechanisms indeed influence a wide variety of affective stimulus–response compatibility phenomena. However, the evaluative coding account does not seem to be sufficient to explain affective S–R compatibility effects. Instead, several studies provide clear evidence in favor of the motivational orientation account that seems to operate independently of evaluative coding mechanisms. Implications for theoretical developments and future research designs are discussed.

A fundamental assumption in emotion research is that emotions predispose the organism to act adaptively in a frequently changing environment. Therefore, many emotion theorists postulate a close link between emotion and action tendencies (cf. Darwin, 1872; Frijda, 1986). In order to meet the requirements of survival, appropriate responses to significant stimuli in the environment must be selected. From an evolutionary perspective, proper response selection should enhance rather than diminish the organism’s fitness. Therefore, the detection of basic principles that could explain how the cognitive apparatus meets the challenge to respond properly has a long tradition in psychological research.

In this sense a vast body of researchers postulated that significant stimuli activate specific motivational orientations that prepare the organism to act adaptively: positive stimuli were assumed to activate approach motivational circuits which in turn trigger approach-related behavioral tendencies, whereas negative stimuli should activate avoidance motivational circuits which trigger avoidance-related behavioral tendencies (Davidson et al., 1990; Gray, 1990; Lang et al., 1990; Strack and Deutsch, 2004). However, recent studies seriously questioned this assumption (Lavender and Hommel, 2007; Eder and Rothermund, 2008). According to Lavender and Hommel (2007) it is not the motivational orientation that mediates the link between evaluative processes and the activation of approach/avoidance responses but rather a simple evaluative coding mechanism. Hence, Lavender and Hommel argued that models accounting for mechanisms of general action control more economically explain why positive (negative) stimuli seem to activate approach-related (avoidance-related) behavior. In what follows, we first present these two alternative theoretical models—the motivational orientation versus the evaluative coding account—in detail. Afterward, we review the literature of different types of approach/avoidance behavior in order to discuss to what extent the recommendation of Lavender and Hommel is applicable.

## Motivational Orientation Account

Emanating from the principle of hedonism, approaching pleasure and avoiding pain are conceived as the most fundamental motivators of human beings (Davidson et al., 1990; Gray, 1990; Lang et al., 1990). In line with this consideration, it is assumed that evaluative processes and approach/avoidance behavior are closely connected (Neumann et al., 2003). Specifically, the Reflective-Impulsive Model (RIM, Strack and Deutsch, 2004), which postulates that behavior is influenced by both impulsive and reflective mechanisms, predicts that the link between evaluation and response activation would be mediated by motivational approach or avoidance orientations. Positive stimuli were assumed to activate the appetitive motivational system and thereby facilitate any kinds of approach behavior, whereas negative stimuli should activate the defensive motivational system and thereby facilitate any kinds of avoidance behavior^1^.

How can this link between evaluation and response activation be described? Within the domain of motivational orientation accounts two different theoretical sub-accounts have been discussed. According to the *specific muscle activation hypothesis* there is a hard-wired link between stimulus evaluations and specific motor responses. Considering arm movements positive stimuli should automatically activate arm flexion whereas negative stimuli should activate arm extension (Cacioppo et al., 1993; Chen and Bargh, 1999). On the contrary, there has also been proposed a *distance regulation hypothesis* to describe the link between evaluation and behavior which assumes that it is not a particular muscle movement that is activated by valenced stimuli but rather a certain motor response that contextually effectuates approach or avoidance (Markman and Brendl, 2005; Seibt et al., 2008). In this sense, the distance regulation hypothesis implies that the link between evaluation and behavior is flexible as a function of contextual factors whereas the specific muscle activation hypothesis assumes the link to be inflexible.

### Empirical Evidence

First, evidence for the assumption that positive stimuli would trigger motivational approach and negative stimuli would trigger motivational avoidance came from a seminal study by Solarz (1960). In this study participants were required to respond to the valence of stimulus words fixed on a movable stage by pushing the stage away from (i.e., avoidance movement), or pulling it toward (i.e., approach movement), them. Overall participants were faster in approaching positive compared to negative words and avoiding negative compared to positive words. This basic affective stimulus–response compatibility effect was later replicated by Chen and Bargh (1999) who reasoned that “approach-like muscle movements” were faster in response to positive stimuli whereas “avoidance-like muscle movements” were faster in response to negative stimuli. Therefore, it has been assumed that the appetitive motivational system would automatically trigger arm flexion whereas the defensive motivational system would trigger arm extension (see also Cacioppo et al., 1993).

However, the idea that specific muscle movements were hard-wired or inflexibly linked to different motivational circuits has been challenged. Markman and Brendl (2005) as well as Seibt et al. (2008) convincingly demonstrated that the direction of the affective S–R compatibility effect is affected by the reference frame induced via task instruction. In both the Solarz (1960) and the Chen and Bargh (1999) studies participants were required to move a lever toward or away from themselves (i.e., subject-based instructions). In doing so, lever movements toward (away from) their own body were faster in response to positive (negative) stimuli. Conversely, when an object-based instruction (*Move the lever toward or away from the stimulus!*) was given (as for example in Seibt et al., 2008) opposite compatibility effects could be observed: lever movements toward (away from) their own body were faster in response to negative (positive) stimuli (see also Laham et al., 2014). Therefore, different motivational circuits do not seem to directly trigger specific approach- or avoidance-like muscle movements (e.g., arm flexion or extension) but rather those movements that are interpreted as approach or avoidance. In sum, the evidence so far seems to favor the distance regulation hypothesis as a specification of the motivational orientation account.

### Automaticity of the Link between Evaluative Processes and Approach/Avoidance Tendencies

A vast body of literature has repeatedly shown that humans automatically^2^ evaluate stimuli they face (see Bargh, 1997, for an overview; Zajonc, 1980). Thus, the affective meaning of a wide variety of stimulus classes (pictures, facial expressions, words, odors etc.) seems to be extracted automatically. Acting on the assumption that evaluative processes and approach/avoidance action tendencies were closely linked to each other one would expect that valenced stimuli automatically prime specific action tendencies. Therefore, any positive stimulus is supposed to automatically trigger approach behavior whereas any negative stimulus should automatically trigger avoidance responses. Indeed, a few studies demonstrated that affective stimulus–response compatibility effects also occur in experimental settings where participants were either instructed to respond to a non-valence stimulus feature (cf. Seibt et al., 2008) or to respond by pushing versus pulling a lever whenever a stimulus appeared on the screen (cf. Chen and Bargh, 1999, Experiment 2; Duckworth et al., 2002, Experiment 3). On the other hand, several other studies failed to show affective stimulus–response compatibility effects when valence was not a task-relevant feature (Rotteveel and Phaf, 2004; Lavender and Hommel, 2007). However, comparability of these studies is limited due to overall differences in their experimental parameters. Therefore, Phaf et al. (2014) reported results of a meta-analysis conducted on numerous manual reaction time studies where approach/avoidance movements were to be made in response to valenced stimuli. Interestingly, the authors observed a medium-sized affective S–R compatibility effect in studies where participants explicitly had to categorize stimulus valence. However, there was no such affective compatibility effect at all when valence was task-irrelevant. This finding might question the notion that affective stimuli automatically trigger action tendencies of approach or avoidance. However, Laham et al. (2014) recently published another meta-analysis based on other criteria for selecting studies to integrate in the analysis. Remarkably, this study did not reveal any significant influence of evaluation goals on the affective compatibility effect (although their results tended in the same direction as those reported by Phaf et al., 2014). Hence, there seem to be studies that provide evidence in favor of the automaticity hypothesis whereas others do not do so.

Having a closer look on literature on different types of approach-avoidance tasks, it becomes obvious that the degree of automaticity (in terms of independency of evaluation intentions) might vary across different task settings. Krieglmeyer and Deutsch (2010) contrasted three types of tasks. First, they used the manikin paradigm, where participants were instructed to imagine being a manikin presented on the screen (De Houwer et al., 2001, Experiment 4; Krieglmeyer et al., 2010, 2011, 2013). This manikin has to be moved toward or away from valenced stimuli via button presses. Second, the regular joystick paradigm has been used where participants were instructed to push or pull the joystick in response to valenced stimuli. Third, a feedback-version of the regular joystick task has been introduced where pushing and pulling the joystick always resulted in a visual zooming effect so that the stimulus either appears to come closer of disappear after responding. A direct comparison of these three types of approach-avoidance tasks revealed that the affective compatibility effect occurred independently of the goal to evaluate the stimuli only when participants performed the manikin or the feedback-joystick task, i.e., those versions of the approach-avoidance task that provided a clear approach- or avoidance-related visual feedback. Therefore, whether an action is interpreted as an approach or avoidance movement depends on the perceivable action consequences. As perceivable action effects seem to play a crucial role in automatic activations of approach-avoidance behavior Van Dantzig et al. (2008) proposed to conceive approach- and avoidance-related action tendencies as “flexible action plans that are represented in terms of their effects” (p. 1298).

## Evaluative Coding Account

So far we have seen that the existence of perceivable approach- or avoidance-related action effects seems to be a necessary precondition for automatic occurrence of affective stimulus–response compatibility effects (Van Dantzig et al., 2008; Krieglmeyer and Deutsch, 2010). However, the idea that action effects are an important determinant of the activation of motor responses is not new. Indeed, in the field of cognitive psychology anticipated action consequences are intensively discussed to be involved in the generation of any motor response (Prinz, 1997; Hommel et al., 2001; Kunde et al., 2007). Following the argumentation of Lavender and Hommel (2007), for this reason affective and non-affective stimulus–response compatibility phenomena share so many basic characteristics that one should seriously doubt that both emanate from different mechanisms. In fact, Lavender and Hommel suggest that both perceptual (for example, the Simon effect; Simon, 1990; Lu and Proctor, 1995) and affective S–R compatibility effects can be explained by a general framework accounting for perception–action interactions, namely the theory of event coding (TEC; Hommel et al., 2001). Deriving from ideomotor principle (Lotze, 1852; Harless, 1861; James, 1890) the TEC postulates that perceived stimuli and response features (including their perceptual action effects) were coded as structurally identical event codes in a common representational domain. Therefore, it is assumed that actions were represented in terms of their anticipated consequences. Furthermore, specific actions can be primed as a result of feature overlap. That is, if a stimulus and an action share specific features, responses would be faster.

Eder and Rothermund (2008) applied this logic to affective S–R compatibility effects. Thus, on the one hand, valence can be considered as one stimulus feature (among others) either being coded as *positive* or *negative*. On the other hand, in affective S–R compatibility paradigms participants were provided with specific action goals. Approach movements can be considered as responses *toward* the self/object whereas avoidance movements can be considered as responses *away from* the self/object. Hence, these response codes themselves carry a specific valence so that a *positive* stimulus code might trigger the goal to respond *toward* a reference point whereas a negative stimulus code might trigger the goal to respond *away from* a reference point. Indeed, Eder and Rothermund (2008) convincingly demonstrated that the affective S–R compatibility effect can be interpreted as a compatibility effect between stimulus valence and response label valence. Responses to positive stimuli were faster when introduced with a positive response label (such as: Move the joystick *up* or *toward* the self/object!). On the contrary, responses to negative stimuli were faster when introduced with a negative response label (such as: Move the joystick *down* or *away from* the self/object!). Consequently, a simple feature overlap mechanism as postulated by the TEC can account for affective stimulus–response compatibility effects.

Accordingly, in terms of the TEC, valence is considered as only one stimulus feature among others (such as color and shape). Accepting this theory to entirely account for the observed affective compatibility phenomena reported in the literature, one must deduce that it is not the motivational orientation that mediates the link between evaluative processes and response activation but rather a simple feature comparison mechanism. On the basis of this argumentation, Lavender and Hommel (2007) suggest “exploring the possibility of explaining all compatibility phenomena within the same theoretical framework—and only construct separate models if this attempt turns out to fail” (p. 1293).

## Contrasting Motivational Orientation vs. Evaluative Coding

In the literature there are two different theoretical approaches that try to explain the mechanisms underlying affective stimulus–response compatibility phenomena. The motivational orientation account assumes that approach and avoidance motivational systems mediate the link between stimulus evaluation and response activation. Within this domain, several authors argued that these motivational systems are linked to specific muscle groups so that positive stimuli would prepare the organism to respond with approach-like motor movements (e.g., arm flexion) whereas avoidance-like motor movements (e.g., arm extension) were activated by negative stimuli (*specific muscle activation hypothesis*; Cacioppo et al., 1993; Chen and Bargh, 1999). However, other researchers convincingly argued that affective stimuli not necessarily activate specific muscle groups but rather those responses that are situationally interpreted as approach or avoidance for example due to their perceivable action effects (*distance regulation hypothesis*; Markman and Brendl, 2005; Seibt et al., 2008; Van Dantzig et al., 2008). Until now, evidence for the specific muscle activation hypothesis seems to be rather weak.

On the contrary, in recent years it has been argued that general theories of human motor control might be sufficient to explain non-affective as well as affective S-R compatibility phenomena (Lavender and Hommel, 2007; Eder and Rothermund, 2008). Therefore, it has been proposed an evaluative coding account of affective S-R compatibility effects (Eder and Rothermund, 2008) in which it is assumed that positive (negative) stimuli activate positively (negatively) connotated responses due to feature overlap. Hence, several researchers deny that motivational processes would mediate the link between evaluation and behavior.

But which of these two theoretical approaches is more suitable to account for affective stimulus–response compatibility phenomena? Until now, there are only a few published studies that tested these theoretical accounts against each other. Laham et al. (2014) recently conducted a meta-analysis across 68 studies that examined affective S–R compatibility effects. In a multiple regression analysis it has been tested whether response labels and/or motivational framing would be significant predictors of the effect sizes. Interestingly, the authors reported that response labels had a significant influence whereas motivational framing does not. Thus, it has been concluded that “to the extent that motivational framing disambiguates action meaning it does so via assigning affective labels to responses” (Laham et al., 2014, p. 16).

However, in adopting the manikin paradigm Krieglmeyer et al. (2010) tried to directly contrast the motivational orientation and the evaluative coding account in an empirical study. In a series of experiments, the manikin was presented either above or below a centrally presented valenced word. The task was to move the manikin up or down depending on the word valence (Experiment 1) or the lexical category (Experiment 2 and 3). On the one hand, the evaluative coding account predicted that positively labeled responses (i.e., moving the manikin *upward*) should be faster in response to positive words whereas negatively labeled responses (i.e., moving the manikin *downward*) should be faster in response to negative words. On the other hand, the motivational orientation account predicted that moving the manikin toward positive and away from negative words should be faster than moving it in the reverse direction. With such an experimental setup, Krieglmeyer et al. (2010) provided evidence in favor of the motivational orientation account: responses to positive words were faster when they decreased the distance between the manikin and the stimulus whereas responses to negative words were faster when they increased the distance between the manikin and the stimulus. Most importantly, at least in two of the three experiments, this effect was independent of evaluative compatibility between stimulus valence and response label valence. Therefore, this study provided evidence for parallel running of both mechanisms.

In sum, there are numerous studies that repeatedly revealed an influence of evaluative coding mechanisms on affective stimulus–response compatibility effects (Eder and Rothermund, 2008; Krieglmeyer and Deutsch, 2010; Laham et al., 2014). On the contrary, the results concerning the influence of motivational orientations on response activation seem to be rather mixed. On the one hand, in the meta-analysis by Laham et al. (2014) the influence of motivational orientations on the affective compatibility effect disappeared when evaluative coding processes have been controlled. On the other hand, Krieglmeyer et al. (2010) clearly provided evidence in favor of the motivational orientation mechanism that seems to operate independent of evaluative coding processes. However, one major limitation of the results of the meta-analysis is the fact that several important studies (e.g., the one by Krieglmeyer et al., 2010) were excluded on the basis of specific selection criteria. Therefore, it can be reasoned that depending on the task affordances both mechanisms might contribute to affective S–R compatibility effects.

### Effector as Determinant

Taking a closer look at studies on approach/avoidance behavior it becomes obvious that most of the studies reviewed so far exclusively focused on manual responses. The spectrum ranges from lever movements (Chen and Bargh, 1999; Eder and Rothermund, 2008) over object movements (Lavender and Hommel, 2007) to button presses/releases (Wentura et al., 2000; Seibt et al., 2008). Hence, approach and avoidance is operationalized as an arm movement toward or away from a reference point or an arm movement that leads to an approach- or avoidance-related action effect (as for example in the manikin paradigm). However, when analyzing whether affective compatibility phenomena would be mediated by specific muscle activations, distance regulation processes or rather evaluative coding mechanisms only focusing on one specific effector of approach-avoidance responses might lead to biased interpretations of results.

Thinking of a human being that is acting in an environment, of course, the notion that object valence and specific arm movements were linked in a hard-wired fashion (Cacioppo et al., 1993; Chen and Bargh, 1999) makes little sense given that decreasing the distance between the self and an object can be achieved via arm extension or flexion depending on whether the object is already held in hand or not. Therefore, whether an arm movement can be interpreted as approach or avoidance must be extremely context dependent in order to efficiently act in a changing environment. On the one hand this might have biased interpretations toward the distance regulation hypothesis as compared to the specific muscle activation hypothesis. On the other hand, this context dependency paired with the inevitable flexibility of arm movements might also promote evaluative coding mechanisms. Since the type of approach-avoidance task seems to be an important factor (cf. Krieglmeyer and Deutsch, 2010) it is also conceivable that the effector of approach-avoidance responses plays a crucial role in the debate on whether motivational orientation accounts or the evaluative coding approach would be an appropriate theoretical framework to account for affective S–R compatibility effects.

#### Whole-body Movements as Approach/Avoidance Responses

The studies reviewed so far have one important aspect in common: in all of them approach and avoidance have been conceptualized in terms of changes in physical distance. However, it can be argued that unimanual arm movements may not be the ideal instantiations of responses initiated to increase or decrease the distance between a stimulus and the self because basic action consequences that naturally occur when an individual increases or decreases the physical proximity between the self and a stimulus (e.g., changes in visual angle) fail to appear. Hence, Stins et al. (2011) introduced whole-body movements as an alternative measure of approach/avoidance responses (see also Koch et al., 2009). In their study participants had to step in a forward (approach) or backward direction (avoidance) in response to the valence of facial expressions presented on a computer screen. The authors observed faster response initiation in stimulus–response compatible, compared to incompatible, conditions. Similarly, Stins and Beek (2011) replicated this compatibility effect for whole-body movements using pictures of emotional scenes as stimuli.

Moreover, a recent study by Ly et al. (2014) provides evidence that even whole-body approach/avoidance responses were automatically triggered by valenced stimuli. The authors presented gems on the left or right side of the screen which were preceded by a task-irrelevant facial expression. Participants were instructed to step sideways either toward (approach) or away from the gem (avoidance). Ly et al. showed that whole-body approach (avoidance) movements could be initiated faster when preceded by a positive (negative) facial expression. In sum these results seem to further support Stins et al. (2011) who reasoned that whole-body movements might be a much “more direct measure of approach/avoidance behavior” (p. 604) with high ecological validity because with this alternative effector quite similar affective stimulus–response compatibility effects could be observed.

How can these affective stimulus–response compatibility effects for whole-body movements be explained? Typically researchers who investigated whole-body approach/avoidance responses (Stins and Beek, 2011; Stins et al., 2011; Ly et al., 2014) explain these compatibility effects in terms of the motivational orientation account. Due to this account affective stimuli should activate compatible motivational circuits so that any kind of appropriate behavior is primed. Thus, positive (negative) stimuli automatically activate the approach (avoidance) motivational system triggering approach-related (avoidance-related) movements. Considering a step forward (i.e., toward a stimulus) as an approach movement and a step backward (i.e., away from a stimulus) as avoidance, the motivational orientation account seems to provide an appropriate theoretical framework to explain whole-body affective S–R compatibility effects.

But is the specific muscle activation hypothesis or rather the distance regulation hypothesis an appropriate theoretical model to specify the motivational orientation account? We argue that the processing of affective stimuli must be flexibly linked to the activation of whole-body movements. First, from an evolutionary perspective, it makes little sense that a negative stimulus, such as a venomous snake, should always activate a tendency to step backward, because it clearly depends on the position of the snake whether a step backward or forward would be the optimal response to ensure survival. Second, the results reported by Ly et al. (2014) clearly demonstrated that it is not a specific movement that is activated by valenced stimuli. As already mentioned in this study a centrally presented emotional face appeared as a prime on a screen in front of the participant, following which, whole-body approach/avoidance movements were to be made in response to non-valent targets appearing on the left or right side of the screen. These approach/avoidance movements were implemented as steps to the left or right instead of steps in the forward or backward direction. The authors observed that whether a step to the left or to the right was triggered by positive or negative stimuli depended on the location of the target. Therefore, situational factors must have influenced whether a movement is interpreted as approach or avoidance.

However, whole-body affective S–R compatibility phenomena can also be explained in terms of the TEC (Hommel et al., 2001). According to the evaluative coding account responses should be speeded if stimuli and responses assigned to them share certain features. Taking a closer look at studies of whole-body approach/avoidance movements (Stins and Beek, 2011; Stins et al., 2011; Ly et al., 2014) it becomes obvious that in all of these studies movement direction and the affective connotation of response labels assigned to these movements were confounded: approach movements were always introduced as responses *toward* the stimulus whereas avoidance movements were always introduced as responses *away from* the stimulus. Therefore, the compatibility effects reported in the literature can easily be explained as a consequence of feature overlap (cf. Eder and Rothermund, 2008). Furthermore, empirical evidence for a flexible link between affective stimuli and whole-body movements again stems from Ly et al. (2014). In this study the emotional prime stimulus appeared in front of the participants following which sideways steps toward or away from the target had to be executed. Again, sideways steps *toward* the target stimulus could be initiated faster when preceded by a positive facial expression whereas sideways steps *away from* the target could be initiated faster when preceded by a negative facial expression. However, in one aspect such an effect is contradictory with the predictions derived from the distance regulation hypothesis because a step to the left as well as a step to the right after a centrally presented emotional stimulus always physically increases the distance between the self and the position where the prime stimulus appeared. Therefore, it is conceivable that it was the framing of responses as *toward* or *away* that produced the observed affective S-R compatibility effects for whole-body movements.

Although the motivational orientation account seems to be favored (Stins and Beek, 2011; Stins et al., 2011; Ly et al., 2014), as far as we know, until now there has been no study that directly tested whether whole-body affective S–R compatibility effects can be explained in terms of the evaluative coding or motivational orientation account. One might argue that the mechanisms underlying whole-body movements might be similar to the mechanisms underlying arm movements, as both effectors target chances in physical proximity between the self and a stimulus. But, until now there is no study that tested these two accounts against each other.

#### Facial Muscle Contractions as Approach/Avoidance Responses

The literature we have reviewed so far defined approach and avoidance in terms of regulation of physical distance. However, approach and avoidance orientation might also be defined in terms of regulation of the social distance. Therefore, in addition to arm or whole-body movements, there are several other forms of recordable behavioral response channels that can be viewed as an indication of approach or avoidance; for example, horizontal versus vertical head movements (Wells and Petty, 1980; Förster and Strack, 1996), reflexes (Lang et al., 1990) or facial muscle contractions (Neumann et al., 2005). Given that arm movements (as well as whole-body movements, too?) seem to be extremely context dependent (Markman and Brendl, 2005; Eder and Rothermund, 2008; Seibt et al., 2008) it can be supposed that neither arm nor whole-body movements are the ideal operationalization of approach and avoidance orientations. Instead, the most critical test for the valence–approach/avoidance tendency link would therefore be the analysis of a response channel that unambiguously reflects approach versus avoidance orientation. According to Neumann et al. (2003) “facial expressions meet this criterion … [because] it does not depend on the point of reference that a smile reflects an approach and a frown an avoidance orientation” (p. 376). Moreover, facial muscles are controlled by both cortical and sub-cortical pathways (Rinn, 1984). This suggests that both goals and evaluative processes might have an impact on facial muscles.

Reviewing the literature of facial muscle contractions it becomes obvious that there has recently been developed an experimental paradigm that is comparable to manual or whole-body approach/avoidance paradigms. This paradigm is based on voluntary facial muscle contractions recorded via electromyogram (EMG). In prototypical studies participants were required to voluntarily contract specific muscles of the face in response to significant stimuli (Dimberg et al., 2002; Neumann et al., 2005, 2014a; Kunde et al., 2011; Otte et al., 2011). Analyses mainly focus on mean response latencies with which specific muscles can be contracted whereas response latency is defined as the time from stimulus onset until response onset (cf. Chiew and Braver, 2010) or until the signal reaches a specific proportion of individual maximum muscle activity (cf. Neumann et al., 2014a).

In adopting this facial response paradigm several researchers provided further evidence for the assumption that positive stimuli would trigger approach whereas negative stimuli would trigger avoidance. In a study by Dimberg et al. (2002) participants were instructed to voluntarily contract either their *zygomaticus major* (i.e., muscle that raises the corners of the mouth producing a smile) or their *corrugator supercilii* (i.e., muscle that pulls the eyebrows together producing a frown) in response to positive and negative facial expressions (Experiment 1) or pictures (Experiment 3). The authors reported that contractions of the zygomaticus muscle could be initiated more quickly in response to positive stimuli and contractions of the corrugator muscle could be initiated more quickly in response to negative stimuli. This result was later replicated by Neumann et al. (2005) using positive and negative words as stimuli. Thus, research on facial muscle contractions revealed an affective stimulus–response compatibility effect comparable with research on manual or whole-body responses.

However, in contrast to manual S–R compatibility phenomena, the link between stimulus valence and facial approach/avoidance behavior seems to be much more automatic because several researchers repeatedly observed an affective S–R compatibility effect when participants performed a valence-irrelevant task (Neumann et al., 2005; Otte et al., 2011). In Experiment 2 of the Neumann et al. (2005) study participants were simply instructed to contract either the zygomaticus or the corrugator muscle whenever a word appeared on the screen. The task did not require them to evaluate these stimuli. Similarly, in the Otte et al. (2011) study voluntary zygomaticus and corrugator muscle contractions were to be made in response to the gender of stimulus persons showing different emotional expressions. In both studies, zygomaticus (corrugator) responses were faster in response to positive (negative) stimuli although valence was clearly task-irrelevant.

Further evidence for an automatic activation account stems from studies recording spontaneous muscle activity when participants perceived valenced stimuli. For example, Dimberg et al. (2000) demonstrated an increased activity over zygomaticus major when participants were unconsciously exposed to positive facial expressions whereas activity of the corrugator supercilii increased when participants were unconsciously exposed to negative facial expressions. Moreover, simply viewing positive pictures leads to increased activity of the zygomaticus major whereas viewing negative pictures increases the activity of the corrugator supercilii (Lang and Bradley, 2007). Consequently, valenced stimuli seem to automatically trigger facial approach/avoidance responses even in the absence of an intention to evaluate.

How can these affective S–R compatibility effects for facial responses be explained? Recent research suggests that affective stimulus–response compatibility phenomena can be explained within the TEC (Lavender and Hommel, 2007; Eder and Rothermund, 2008). First evidence for the assumption that feature overlap mechanisms even influence the activation of facial muscles comes from a study by Kunde et al. (2011). In this study voluntary zygomaticus versus corrugator contractions were to be made to indicate the color of dots appearing on the screen. Immediately after responding a facial expression was presented in the center of the screen that served as an action effect. In one experimental block the facial expression was always compatible with the response shown by the participant (i.e., a smiling face after zygomaticus response or frowning face after corrugator response) whereas in the other block the facial expression was always incompatible (i.e., a smiling face after corrugator response or frowning face after zygomaticus response). Therefore, responses which had noticeable features (e.g., feeling the corners of the mouth rising up) always resulted in a perceivable action effect. In terms of the TEC it is assumed that overlap between certain stimulus and/or response features leads to faster responses. Therefore, one would expect that facial responses should be faster when anticipating a compatible, compared to an incompatible, action effect to occur. Indeed, Kunde et al. (2011) demonstrated faster responses in response–effect compatible, compared to response–effect incompatible, conditions. Consequently, anticipated response consequences seem to be integrated in the activation of facial muscles.

Whether the TEC can also entirely account for facial affective stimulus–response compatibility effects has recently been investigated by Neumann et al. (2014a). In this study the experimental logic introduced by Eder and Rothermund (2008) has been adapted to facial responses. Thus, responses were introduced using positive versus negative response labels. Participants were instructed to classify stimulus words due to their valence by either showing the “sun”-response (i.e., positive response label) or the “rain”-response (i.e., negative response label). Half of the participants were required to respond with joystick movements away or toward themselves (i.e., manual responses) whereas the other half were required to respond with voluntary contractions of the zygomaticus or corrugator muscle (i.e., facial responses). For manual responses Neumann et al. (2014a) replicated the results reported by Eder and Rothermund (2008): there was a pronounced compatibility effect between stimulus valence and response label valence indicating faster “sun”-responses to positive words and faster “rain”-responses to negative words irrespective of the arm movement that was required. This result further confirms the assumption that feature overlap mechanisms contribute to affective stimulus–response compatibility effects as observed for arm movements. However, for facial responses the compatibility effect between stimulus valence and response label valence was much weaker compared to manual responses. In fact, Neumann et al. (2014a) reported having found a pronounced stimulus valence–muscle compatibility effect indicating faster responses to positive words with the zygomaticus muscle and faster responses to negative words with the corrugator muscle. This compatibility effect was slightly reduced when response label valence mismatched the respective muscle (i.e., zygomaticus = “rain”-response; corrugator = “sun”-response). Therefore, feature overlap does influence the activation of facial responses (cf. Kunde et al., 2011), too, but the results reported by Neumann et al. (2014a) strongly suggest that affective stimulus–response compatibility effects as observed for facial muscle movements cannot entirely be explained via a simple feature overlap mechanism as postulated by the TEC.

Can we conclude that the observed affective stimulus–response compatibility effects for facial muscle movements are mediated by motivational orientations? One alternative mechanism that could potentially account for the observed response pattern is automatically activated mimicking behavior. It is well known that the perception of an emotional expression automatically leads to imitation processes in the observer. Thus, a smiling face automatically triggers activation of the zygomaticus muscle whereas a frowning face triggers corrugator activity (Dimberg et al., 2000; Likowski et al., 2008, 2011). Such an account especially applies for studies using facial expressions as stimuli (Dimberg et al., 2000, 2002, Experiment 1; Otte et al., 2011). However, this objection has to deal with the vast body of literature demonstrating exactly the same response pattern using pictures (Dimberg et al., 2002, Experiment 3; Lang and Bradley, 2007) or even words as stimuli (Neumann et al., 2005, 2014a). Furthermore, Neumann et al. (2014b) recently provided evidence for the assumption that mimicking behavior might also be an evaluative process rather than an imitation mechanism. Amongst other muscles the authors recorded spontaneous muscle activity over levator labii (i.e., muscle that wrinkles the nose producing an expression showing disgust) in response to facial expressions. Interestingly, contraction of the levator labii in a stimulus person did not automatically trigger activation of the levator labii in the observer which could have been regarded as an indicator of motor-mimicry. Instead the perception of a facial expression of disgust leads to increased activity over the corrugator muscle which can rather be seen as a fast evaluative response to facial displays of others. Therefore, we think that imitation mechanisms do not provide a sufficient explanation for automatic responses to shortly presented emotional displays.

If motivational orientations mediate the link between stimulus evaluation and facial muscle contractions is the specific muscle activation or rather the distance regulation hypothesis an appropriate specification? In other words, are certain facial muscles inflexibly linked to motivational approach and avoidance circuits? Empirical evidence for this assumption stems from studies on cerebral asymmetry (Davidson et al., 1990; Allen et al., 2001; Coan et al., 2001). According to Davidson (1984, 1987, 1992) motivational orientations involve asymmetrical activations of the anterior cortical regions in the way that the left anterior region subserves the approach motivational systems whereas the right anterior region subserves the avoidance motivational system. Interestingly, the degree of cerebral asymmetry seems to be associated with the activation of specific facial muscle groups. In a study by Davidson et al. (1990) participants watched positive and negative film clips while recording EEG activity and videotaping their facial expressions. The authors observed greater left compared to right frontal activation during those time frames where subjects showed facial expressions of happiness whereas greater right as compared to left frontal activation occurred when subjects showed facial expressions of disgust. This link between motivational orientations and facial muscle contractions seems to be bidirectional. A causal influence of motivational orientations on the activation of facial muscle groups has been shown by Allen et al. (2001). They used biofeedback training to increase the relative left or right-sided activation of the frontal cortex. With this method they successfully manipulated the degree of frontal asymmetry. Afterward, facial muscle activation has been recorded with EMG while participants watched different film clips. The authors observed that increasing relative right-sided activation with biofeedback decreased activation of the zygomaticus major muscle whereas increasing relative left-sided activation decreased activation of the corrugator supercilii muscle. In addition to that it has been shown that manipulations of facial expressions also influence cortical activity in the anterior regions. In a study by Coan et al. (2001) the directed facial action task was used in which subjects were instructed to voluntarily adopt certain facial expressions while EEG activity was recorded. It could be demonstrated that adopting negative facial expressions resulted in relatively less left frontal activation than adopting positive facial expressions. All these findings are hardly explainable by a flexible link between motivational orientations and facial smuscle activation. In fact, we conclude that this link is rather hardwired so that the specific muscle activation hypothesis seems to provide an appropriate theoretical framework to explain affective S–R compatibility effects as observed for facial muscle responses.

### Integration of Findings

The literature reported above examined different types of effectors of approach/avoidance responses. On the one hand, we reviewed unimanual arm as well as whole-body movements, which are effectors that reflect approach/avoidance in terms of physical distance regulation. We have seen that evaluative coding mechanisms play a crucial role in the activation of manual approach-avoidance responses. This–although not yet tested–might even be the case for whole-body approach-avoidance responses. These results might be seen as support for the notion that affective and non-affective S–R compatibility effects can be integrated into the same theoretical framework (cf. Lavender and Hommel, 2007). However, there are several exceptions were the TEC was not able to entirely account for the observed affective S–R compatibility effects. Instead, for example Krieglmeyer et al. (2010) provided clear evidence that both mechanisms contribute to affective S–R compatibility effects when focusing on the manikin version of the approach-avoidance task.

On the other hand, approach/avoidance can also be conceived as a means to regulate the social distance. In doing so, facial muscles seem to be an appropriate effector of approach/avoidance in the way that they unambiguously reflect specific motivational orientations. Focusing on the literature of facial approach/avoidance responses it becomes even more obvious that both motivational orientation as well as evaluative coding mediate the link between evaluative processes and response activation (cf. Neumann et al., 2014a). Thus, again, evaluative coding is an important mechanism contributing to the well-known affective S–R compatibility phenomena. However, independent of evaluative coding, motivational orientations seem to affect especially those responses that unambiguously reflect approach and avoidance orientations.

In sum, several findings contradict the postulate that one theoretical framework is sufficient to explain all affective as well as non-affective stimulus–response compatibility phenomena (cf. Lavender and Hommel, 2007). Instead, as reasoned by Krieglmeyer et al. (2010), p. 612), “[both mechanisms—the motivational orientation and the evaluative coding mechanism—seem] to operate independently of and in parallel to [each other].” The portion of the variance of the affective S–R compatibility effect that each mechanism might explain seems to vary across different types of approach-avoidance tasks and different effector of approach-avoidance responses.

## Conclusion

Taken together, we have seen that in the literature there are two opposite accounts for affective stimulus–response compatibility phenomena. The motivational orientation account states that valenced stimuli automatically activate corresponding motivational circuits: positive stimuli trigger approach motivation and negative stimuli trigger avoidance motivation (Davidson et al., 1990; Gray, 1990; Lang et al., 1990; Strack and Deutsch, 2004). These motivational orientations in turn trigger corresponding behavioral tendencies resulting in a close link between evaluative processes and approach/avoidance response tendencies. On the one hand, the specific muscle activation hypothesis predicts that motivational orientations inflexibly activate specific muscle contractions. On the other hand, the distance regulation hypothesis predicts that motivational orientations flexibly trigger those responses that situationally result in increased or decreased proximity between the self and an object. The motivational orientation account has recently been challenged by authors arguing that the TEC might also explain affective S–R compatibility effects (Lavender and Hommel, 2007; Eder and Rothermund, 2008). The TEC postulates that response episodes are stored in an event file including codes for each perceivable stimulus and response feature. Any of these features is sufficient to prime responses which share characteristics with that feature. Due to the fact that non-affective and (manual) affective S–R compatibility phenomena share basic characteristics it has been suggested to explain all S–R compatibility effects within the same theoretical framework—namely the TEC (Lavender and Hommel, 2007). The present paper explored to what extent this recommendation is applicable. Indeed, there is substantial evidence suggesting that the TEC is an economical model to explain affective S–R compatibility effects when examining manual and presumably whole-body responses, as well. But studies that adopted a different experimental paradigm as compared to the classical joystick task (e.g., the so-called *manikin task*) provided clear evidence in favor of the motivational-orientation account (Krieglmeyer et al., 2010, 2011, 2013). Moreover, focusing on the literature of approach/avoidance effectors used to regulate the social distance (i.e., facial muscle contractions), it becomes even more obvious that the TEC is not an appropriate theoretical framework to entirely account for the observed congruency effects. Instead of assuming the TEC to entirely account for the observed affective S–R compatibility phenomena, as did Lavender and Hommel (2007), one should rather assume that both mechanisms—the motivational orientation and the evaluative coding mechanism—can operate independently of each other and even in parallel (see also Krieglmeyer et al., 2010).

Furthermore, the effector of approach/avoidance responses seems to be an important determinant. However, until now there has been a lack of studies directly comparing different effectors of approach/avoidance. Similarly, Eder et al. (2013) stated that the “lack of cross-talk within and between different levels of behavioral analysis [within the research domain of approach- and avoidance-motivated behavior] limits scientific insight into more general principles of approach and avoidance motivations, thereby contributing to fragmentation in the field” (p. 228). We think that future research should explicitly differentiate and compare the effectors of approach/avoidance responses in order to counteract this fragmentation. On the one hand, motivational orientation accounts provide a proper theoretical framework to explain the often observed link between evaluative processes and action tendencies. However, the observed affective S–R compatibility effects differ as a function of the effector by which responses were stimulated. Hence, from a theoretical viewpoint it would be an important step forward to refine motivational accounts by including the effector as an important factor in order to gain a deeper insight into the link between evaluative processes and emotional responses.

### Conflict of Interest Statement

The Associate Editor, Fritz Strack, declares that despite being affiliated with the same institution as the author, Ljubica Lozo, the review process was handled objectively and no conflict of interest exists. The authors declare that the research was conducted in the absence of any commercial or financial relationships that could be construed as a potential conflict of interest.

## Footnotes

1 We are aware of the fact that the categorical distinction of positive versus negative stimuli could be regarded as oversimplified. Considering affective stimuli in more detail one might rather characterize them beyond valence, for example, in terms of discrete emotion concepts. In doing so, it becomes obvious that certain stimuli are at least somewhat beyond the scope of approach/avoidance models. Although anger can be viewed as a negatively valenced affect (Carver and Harmon-Jones, 2009) in some studies anger-related stimuli triggered avoidance responses (Marsh et al., 2005; Roelofs et al., 2008), whereas other researchers demonstrated that approach responses were activated by stimuli related to anger (Wilkowski and Meier, 2010). Krieglmeyer and Deutsch (2013) tried to solve this inconsistency by arguing that superordinate goals determine the link between stimulus valence and response activation. Thus, negative stimuli might automatically elicit a motivation to ultimately increase the physical distance irrespective of instantaneous consequences (see also Krieglmeyer et al., 2011). In the case of anger-related stimuli, this goal can be achieved by flight (i.e., a concrete avoidance response) or even by fight (i.e., a concrete approach response) because attacking an opponent likely puts the opponent to flight.

2 Due to Moors and De Houwer (2006) cognitive operations can be described as automatic processes if they are executed unintentional, uncontrolled, goal independent, autonomous, purely stimulus driven, unconscious, efficient, and fast. We were mostly interested in the question of whether the activation of approach-avoidance responses would occur independently of the goal to evaluate stimuli. Therefore, by using the term “automaticity” we refer to the feature of goal independency.
